# Reactive Oxygen Species-Mediated Cellular Stress Response and Lipid Accumulation in Oleaginous Microorganisms: The State of the Art and Future Perspectives

**DOI:** 10.3389/fmicb.2017.00793

**Published:** 2017-05-01

**Authors:** Kun Shi, Zhen Gao, Tian-Qiong Shi, Ping Song, Lu-Jing Ren, He Huang, Xiao-Jun Ji

**Affiliations:** ^1^College of Biotechnology and Pharmaceutical Engineering, Nanjing Tech UniversityNanjing, China; ^2^Jiangsu National Synergetic Innovation Center for Advanced MaterialsNanjing, China; ^3^School of Pharmaceutical Sciences, Nanjing Tech UniversityNanjing, China; ^4^State Key Laboratory of Materials-Oriented Chemical Engineering, Nanjing Tech UniversityNanjing, China

**Keywords:** reactive oxygen species, oleaginous microorganisms, stress response, signaling molecules, lipid accumulation

## Abstract

Microbial oils, which are mainly extracted from yeasts, molds, and algae, have been of considerable interest as food additives and biofuel resources due to their high lipid content. While these oleaginous microorganisms generally produce only small amounts of lipids under optimal growth conditions, their lipid accumulation machinery can be induced by environmental stresses, such as nutrient limitation and an inhospitable physical environmental. As common second messengers of many stress factors, reactive oxygen species (ROS) may act as a regulator of cellular responses to extracellular environmental signaling. Furthermore, increasing evidence indicates that ROS may act as a mediator of lipid accumulation, which is associated with dramatic changes in the transcriptome, proteome, and metabolome. However, the specific mechanisms of ROS involvement in the crosstalk between extracellular stress signaling and intracellular lipid synthesis require further investigation. Here, we summarize current knowledge on stress-induced lipid biosynthesis and the putative role of ROS in the control of lipid accumulation in oleaginous microorganisms. Understanding such links may provide guidance for the development of stress-based strategies to enhance microbial lipid production.

## Introduction

Lipids can be produced by practically all living organisms, and they play pivotal structural and functional roles, including the formation of cell membranes, as well as carbon and energy storage. However, only a few microorganisms, including yeasts, molds, and algae, can accumulate microbial lipids to more than 20% of their dry cell weight (DCW), and thus be defined as oleaginous microorganisms (Donot et al., 2014). Such oleaginous microorganisms are currently emerging as production strains for a variety of applications, and are already significant sources of fatty acid- and lipid derivatives in various industrial fields (Laoteng et al., 2011). The production of microbial lipids is not restricted by season, climate, and location, and can be realized utilizing a variety of inexpensive substrates, such as waste streams from the food industry (Ochsenreitheri et al., 2016). Considering the threat of global warming, the increasing demand for energy, and the foreseeable depletion of easily extracted crude oil, microbial lipids are receiving worldwide attention as a cleaner alternative to fossil fuels. Also, from a dietary point of view, microbial lipids can be a safe, edible substitute for animal fats, which is a traditional source of polyunsaturated fatty acids (PUFAs) (Ratledge, 2004; Ryan et al., 2010; Bellou et al., 2016a). This is particularly significant since PUFAs, such as those belonging to the omega-3 and omega-6 series, are well known for their benefits to human health (Ji et al., 2014, 2015; Xie et al., 2015).

Oleaginous microorganisms are able to accumulate cellular lipids from 25% to an astonishing 70% of their DCW, and this accumulation is normally induced under various environmental stresses, such as high light stress, salt stress, and deprivation of nutrients (Wang et al., 2004; Jeennor et al., 2006; Donot et al., 2014; Fan et al., 2014). Consequently, stress-based strategies are widely used as environmentally friendly approaches to induce lipid overproduction in cultured microorganisms (Sharma et al., 2012), and a wide range of studies were carried out to identify and develop efficient induction techniques for lipid accumulation. Advances provided by the development of “-omics” analysis methods have illuminated the global reorganization of metabolic and transcriptional states and their integration between different stress regimens (Morano et al., 2012; Yang Z.K. et al., 2014; Yu et al., 2017).

On the other hand, oxidative metabolism is the major form of energy production in most living organisms, and reactive oxygen species (ROS) are generated as by-products of various metabolic pathways, including aerobic respiration. It is well established that ROS can inhibit photosynthesis and cause damage to DNA, proteins, lipids. However, over the past two decades, there has been a growing appreciation for the role of ROS as “second messengers” of various signal transduction mechanisms (Schieber and Chandel, 2014; Zhang J. et al., 2016). For example, nicotinamide adenine dinucleotide phosphate (NADPH) oxidases can produce ROS to regulate different cellular processes, such as defense, growth, and acclimation (Lara-Ortiz et al., 2003; Aguirre et al., 2005; Sandalio et al., 2013).

Here we review recent work on stress-induced lipid biosynthesis and the putative role of ROS in controlling lipid accumulation in oleaginous microorganisms. Furthermore, future perspectives on emerging analysis methods and new research directions in the field of ROS signaling are discussed.

## Lipid Accumulation in Oleaginous Microorganisms Under Different Types of Stress

Oleaginous microorganisms produce only small amounts of lipids under favorable environmental conditions, and their lipid biosynthesis machinery can be induced by stress conditions such as nutrient limitation and exposure to damaging physical factors (Chisti, 2007; Georgianna and Mayfield, 2012; Ji et al., 2015).

### Nutrient Limitation

Oleaginous microorganisms can accumulate considerable amounts of lipids under various nutrient limitation conditions, among which nitrogen limitation is recognized as the most successful induction strategy, and is most widely used. Large amounts of omics data had shown some clues of the regulation of carbon flux redistribution to lipids. For example, proteomic analysis demonstrated that nitrogen depletion can upregulate the glycolytic pathway, while the activity of TCA cycle was retarded, thus, leading more carbon flux to fatty acid biosynthesis in *Mucor circinelloides* (Tang et al., 2016). Other types of nutrient starvation that can enhance lipid accumulation include phosphate, silicon, and sulfate limitation (Wu et al., 2011; Ren et al., 2013; Bellou et al., 2016b; Ota et al., 2016).

Generally, two different pathways are involved in lipid synthesis: *de novo* lipid synthesis and *ex novo* lipid synthesis. Fundamental differences on the biochemical level exist between *de novo* lipid synthesis from hydrophilic substrates and *ex novo* lipid synthesis from hydrophobic substrates (Donot et al., 2014). Some studies have reported that nutrient limitation is only effective in the induction of *de novo* lipid synthesis, when growth is carried out on various hydrophilic substances (Papanikolaou and Aggelis, 2011a,b; Donot et al., 2014). Furthermore, nutrient limitation will induce lipid biosynthesis while concomitantly causing growth inhibition (Sajbidor et al., 1990; Nie et al., 2014; Zienkiewicz et al., 2016). However, it is a significant goal to obtain both a great quantity of biomass and a high lipid content for improved lipid productivity. Thus, in practice, oleaginous microorganisms are first cultured in nutrient-rich media for rapid propagation in early process stages, while nutrient limitation is introduced in later stages to induce the overproduction of lipids (Lin et al., 2011; Zhu et al., 2016). For instance, Nie et al. (2014) reported a three-stage fermentation strategy that changes the nutrient components of the medium, which was designed for the efficient production of oil rich in arachidonic acid (ARA) using *Mortierella alpina*.

### Physical Environmental Stresses

The composition and quantity of microbial lipids is species-dependent and can be affected by external environmental factors, such as light intensity, temperature, salinity, dissolved oxygen, etc. (Vitova et al., 2015). Furthermore, in autotrophic microalgae, light capture for photosynthesis is crucial for growth and accumulation of lipid reserves. Thus, adequate light intensity favors the overproduction of microalgal lipids. For example, high light conditions trigger the formation of neutral lipids in the microalgae *Scenedesmus abundans* and *Nannochloropsis* sp., as well as *Botryococcus braunii* and a further *Botryococcus* species (Kojima and Zhang, 1999; Pal et al., 2011; Yeesang and Cheirsilp, 2011; Mandotra et al., 2016).

The incubation temperature has been reported as an important abiotic factor affecting growth and microbial oil accumulation (Laoteng et al., 2011). In *Nannochloropsis oculata*, heat stress rapidly stimulates neutral lipid formation, while raising the temperature up to 25°C causes an elevation of total lipids (Converti et al., 2009). On the other hand, the incubation temperature of oleaginous microorganisms influences their fatty acid composition by changing the desaturase enzyme activity (Amaretti et al., 2010; Xu et al., 2012). James et al. (2013) found that a switch to temperatures lower than 25°C decreased the content of saturated fatty acids in the microalga *Chlamydomonas reinhardtii*. Likewise, the concentration of ARA in *M. alpina* changed from 7.3 to 9.2 g/L, when the temperature changed from 25 to 20°C (Peng et al., 2010).

In some species of microalgae, an increase of intracellular lipid content was observed as a response to osmotic pressure due to salinity stress (Takagi et al., 2006; Yang H. et al., 2014). For example, Duan et al. (2012) reported that *Chlorella vulgaris* experienced a 21.1% increase of lipid yield when exposed to salt pressure. Biosynthesis of lipids is also induced by other physical environmental stresses, such as low oxygen in the microalga *Aurantiochytrium* sp., and dehydration in the green alga *Chlorella kessleri* (Jakobsen et al., 2008; Shiratake et al., 2013).

## Stress-Induced ROS Generation and Its Potential Role in Lipid Accumulation

Since stress-based strategies are widely used as an environmentally friendly approach to microbial lipid overproduction, understanding the relationship between various stress factors and lipid accumulation is of industrial and biotechnological importance (Breuer et al., 2013). With advances in biotechnology and bioinformatics, such as proteomic and genomic analysis, many studies revealed metabolic network shifts toward lipid accumulation under different kinds of stress (Lei et al., 2012; Yang Z.K. et al., 2014; Tang et al., 2016). However, little is known about the link between extracellular stress signals and intracellular lipid synthesis. There may exist potential signal transduction mechanisms that trigger carbon partitioning and lipid accumulation in response to different environmental stresses, which serve to control homeostasis at the cellular level. In recent years, some research results indicated that ROS may be important mediators in this process (Urbano et al., 2014; Yilancioglu et al., 2014).

### Redox Homeostasis and Oxidative Stress

Reactive oxygen species, such as superoxide (O_2_^-^), hydroxyl radical (OH^•^), and hydrogen peroxide (H_2_O_2_) are formed by the partial reduction of oxygen, which is an inevitable aspect of life under aerobic conditions (Montibus et al., 2015). Cellular ROS metabolism is tightly regulated by a battery of biological redox mechanisms, including both antioxidant enzymes and non-enzymatic antioxidant molecules. In a normal physiological state, the level of cellular ROS is in a stable dynamic equilibrium (Zhang J. et al., 2016). However, under specific adverse environmental stimuli, the balance between cellular ROS production and elimination can be disturbed, leading to a locally increased concentration of ROS – i.e., “oxidative stress” (Lushchak, 2011).

Similar to other forms of environmental stress, oxidative stress results in damage of nucleic acids, proteins, and lipids. However, indeed in many eukaryotes, there are well-described mechanisms in which ROS play a key role in the response and adaptation to environmental changes (Bhattacharjee, 2012; Hideg et al., 2013). According to recent reports, ROS may act as decisive signaling molecules of the cellular responses to environmental stresses in oleaginous microorganisms. The relationship between ROS levels and a number of different kinds of stress has been reported, including adverse light conditions, nitrogen depletion, unfavorable salinity, as well as high or low temperature (Shih et al., 2014; Chokshi et al., 2015; Garcia-Rios et al., 2016). In *C. vulgaris*, the specific intracellular ROS level can serve as a general quantitative marker for stress, irrespective of the type of stress induced (Menon et al., 2013).

### Stress Sensing and Putative Concomitant ROS Generation

**FIGURE 1:**
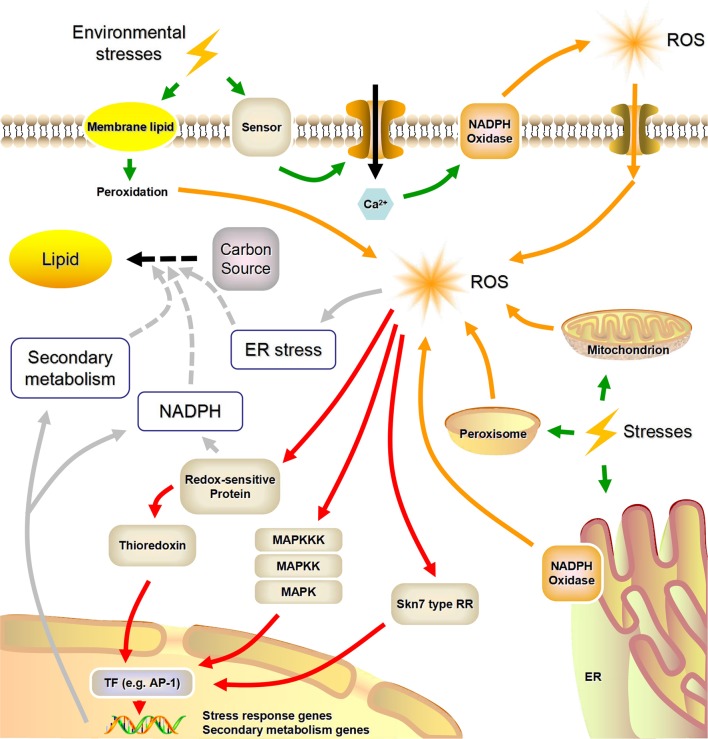
**A possible link between environmental stress factors and lipid biosynthesis mediated by reactive oxygen species (NADPH, nicotinamide adenine dinucleotide phosphate; ROS, reactive oxygen species; MAPK, mitogen-activated protein kinase; MAPKK, MAPK kinase; MAPKKK, MAPKK kinase; TF, transcription factor; RR, response regulator; ER, endoplasmic reticulum; Green arrows, stress sensing; Orange arrows, stress-induced ROS generation; Red arrows, ROS signal transduction; Gray arrows, ROS-induced lipid accumulation).** This figure was modified from Hagiwara et al. (2016) and Zhu (2016).

Eukaryotic microorganisms (e.g., yeasts, molds, and microalgae) possess highly conserved molecular mechanisms that enable them to survive in adverse environments. The machinery encompassing stress sensors, signal transduction and response elements is common to these mechanisms (Lushchak, 2011). In **Figure 1**, we summarized what is known about stress sensing and the putative concomitant ROS generation.

Nicotinamide adenine dinucleotide phosphate oxidases are highly conserved plasma- and endo-membrane enzymes present in animals, plants (including microalgae), and fungi. Some researchers also summarized the functions of certain macromolecules as possible sensors for environmental changes in plants and mammals (Hernandez-Onate and Herrera-Estrella, 2015; Zhu, 2016). Specific signals (e.g., Ca^2+^) that are generated by these stress sensors can lead to the phosphorylation and activation of NADPH oxidases, and activated NADPH oxidases can in turn utilize cytosolic NADPH as the electron donor to reduce extracellular O_2_ to O_2_^-^, causing the subsequent formation of H_2_O_2_ (Herve et al., 2006; Mignolet-Spruyt et al., 2016).

The electron transfer chains (ETC), which exist in mitochondria, endoplasmic reticulum, and peroxisomes, are regarded as the main sources of intracellular ROS (Starkov, 2008; Sandalio et al., 2013). Under certain stimuli, growing ROS concentrations can overwhelm the cellular antioxidant defense systems, causing ROS to be released into the matrix via osmosis (Manoli et al., 2007; Mignolet-Spruyt et al., 2016). Using TEM, Yu et al. (2016) observed mitochondria in *M. alpina* that seemed to be enlarged and less compact than normal with carbon limitation, which in turn may generate ROS. Furthermore, abiotic stresses such as UV and heat instantaneously lead to modifications of the membranes, such as membrane-lipid peroxidation, which are potential sources of ROS. Unlike NADPH oxidase-generated ROS, these membrane-lipid peroxidation products induce non-specific responses to various kinds of environmental stress (Bhattacharjee, 2012).

### Transduction of Intracellular ROS Signals

Compared with other types of ROS, such as O_2_^-^ and OH^•^, H_2_O_2_ is much more stable and can pass membranes through aquaporins, which is an advantage with regard to its signaling capacity (Reczek and Chandel, 2015). The transduction of H_2_O_2_-based signals is mainly centered on sulfur chemistry, with the main player being the reversible oxidative modification of cellular sulfur-containing groups (e.g., cysteine residues and thioredoxin), which in turn results in disturbances of cellular metabolism and signaling pathways in eukaryotic microorganisms (Veal et al., 2007; Scott and Eaton, 2008).

As shown in **Figure 1**, the key part of the adaptive response to H_2_O_2_ is the transcriptional reprogramming of gene expression. The AP-1 family transcription factors are highly conserved among eukaryotes. They are involved in genetic responses to H_2_O_2_ signals associated with the Skn7-type response regulators (Carmel-Harel et al., 2001; Hagiwara et al., 2016). H_2_O_2_ can also oxidize critical cysteine thiol groups of phosphatases in mitogen-activated protein kinase (MAPK) pathways, which appears to be a conserved signaling mechanism for diverse environmental stimuli (Ikner and Shiozaki, 2005; Lushchak, 2011). For instance, the unicellular yeast *Schizosaccharomyces pombe* utilizes a two-component signaling system coupled to a MAPK pathway to perceive and respond to local ROS accumulation (Nguyen et al., 2000; Lara-Rojas et al., 2011). Finally, a number of recent studies have shown that post-translational changes induced by ROS also play an important role in the rapid response to oxidative stress, in addition to the altered global gene expression patterns (Ralser et al., 2009; Morano et al., 2012).

### Possible Links between ROS and Lipid Accumulation

Oleaginous microorganisms mainly accumulate neutral lipids, which account for nearly 90% of their lipid storage (Juneja et al., 2013; Donot et al., 2014). In addition to their traditional and most-studied function in carbon and energy storage, these lipids, and especially PUFAs, may act as antioxidants or otherwise protective defense molecules in the stress response (Hu et al., 2008). For example, the increase of fatty acid unsaturation under low temperature stress has been generally linked to an adaptation mechanism which the lipid-producing fungi use to maintain membrane fluidity (Certik and Shimizu, 1999; Ji et al., 2014). The overproduction of lipids presents an indispensable buffer against stress conditions.

As shown in **Table 1**, improving experimental evidence seems to point in the direction that intracellular ROS may in fact be mediators of lipid accumulation in oleaginous microorganisms (Yilancioglu et al., 2014; Chokshi et al., 2015). A number of studies showed that stress-induced lipid accumulation always goes along with increasing antioxidant defenses (e.g., oxidative-stress response proteins) or increasing intracellular ROS levels (Yang et al., 2013; Yilancioglu et al., 2014). As stress markers, the specific intracellular ROS levels have been found to be linked to the specific intracellular neutral lipid levels in an inverse and direct power law fashion in *C. vulgaris* (Menon et al., 2013). In fact, the proper exposure to exogenous ROS, such as H_2_O_2_, but also certain nanomaterials, can trigger neutral lipid formation (Yu et al., 2015).

**Table 1:** Stress-induced reactive oxygen species (ROS) generation in different oleaginous microorganisms.

Stress factor	Oleaginous microorganism	Lipid production	ROS generation	Reference
Salinity	*Scenedesmus* sp.	33.13% of lipids accumulated under salinity stress (400 mM NaCl).	Higher H_2_O_2_, MDA, APX, and proline contents were observed with an increase of NaCl.	Pancha et al., 2015
High light intensity	*Chlorella* sp. and *Monoraphidium* sp.	Carbon allocation converted from protein and carbohydrate to lipid under high light. Neutral lipid productivity was significantly promoted.	Along with increasing lipid accumulation, ROS-scavenging enzymes also increased.	He et al., 2015
Low temperature	*Scenedesmus* sp.	At low temperatures, the lipid content per microalgal biomass increased.	The ROS levels at 10°C and 20°C were higher than that under higher temperature.	Li et al., 2011
Nitrogen limitation	*Dunaliella salina*	Nitrogen limitation increased cellular lipid content up to 35% under 0.05 mM nitrogen concentration.	Under nitrogen depleted cultivation conditions, higher MDA, CAT, APX, and SOD were observed.	Yilancioglu et al., 2014
	*Nitzschia closterium f. minutissima*	Neutral lipid increased 48, 111, 171, and 216% compared to the control, with nitrogen concentrations of 712, 491, 270, and 159 μM.	Intercellular ROS was enhanced under low nitrogen stress.	Liu et al., 2012
	*Acutodesmus dimorphus*	Nitrogen limitation is effective to produce biomass containing 29.92% of lipid (comprising about 75% of neutral lipid).	Nitrogen limitation resulted in the accumulation of ROS.	Chokshi et al., 2017
	*Mucor circinelloides*	The rate of lipid production goes up after nitrogen exhaustion.	Some proteins involved in signal transduction and redox homeostasis were upregulated upon nitrogen depletion.	Tang et al., 2016
	*Chlorella pyrenoidosa*	The total lipid and neutral lipid contents exhibit the most marked increment under nitrogen deficiency, achieving 50.32 and 34.29% of DCW, respectively.	Nitrogen limitation resulted in the co-occurrence of ROS and lipid accumulation.	Fan et al., 2014


However, only a few possible mechanisms of ROS-mediated lipid accumulation have been illuminated, and direct experimental evidence is still absent. Some hypotheses surrounding the mechanisms of ROS-dependent lipid accumulation are shown in **Figure 1**. As lipids are highly reduced molecular species, neutral lipid overproduction requires large quantities of NADPH, whose primary sources are the oxidative pentose phosphate pathway and malic enzyme (Wasylenko et al., 2015). In what appears to be a common mechanism, within seconds of oxidative stress the carbon metabolic flux changes from glycolysis to the oxidative pentose phosphate pathway by post-translational modification of glycolytic enzymes (Ralser et al., 2009). On the other hand, the stored neutral lipids in oleaginous microorganisms are deposited within lipid droplets, and there is evidence that endoplasmic reticulum stress is an activator of lipid droplets formation (Jacquier et al., 2011). Since ROS are a well-known trigger of endoplasmic reticulum stress, this is another possible mechanism by which ROS stress enhances the formation of lipid droplets (Yu et al., 2015). Moreover, previous studies have demonstrated that some transcription factors, such as AP-1, which are associated with the oxidative-stress response, also participate in a regulatory network that induces secondary metabolism (Montibus et al., 2015). Considering the crosstalk between lipid biosynthesis and secondary metabolism, this appears to be another potential link between intracellular ROS signaling and lipid metabolism (Reyes et al., 2014; Zhang Z. et al., 2016).

Furthermore, the overall level of intracellular ROS cannot be ignored. The generation of low levels of ROS by environmental stresses initiates adaptive responses, including lipid biosynthesis and accumulation. By contrast, high levels of ROS may cause cellular damage, leading to the consumption of stored lipids as energy source in order to maintain cellular homeostasis. It has also been reported that the external addition of antioxidants, such as sesamol, ginsenosides, and ascorbic acid, can scavenge intracellular ROS and enhance PUFA content in oleaginous microorganisms (Liu et al., 2015; Ren et al., 2017).

## Conclusion and Perspectives

Microbial lipid accumulation can be regarded as a buffer against environmental stresses, such as nutrient limitation and adverse physical environments. Stress-based lipid production strategies have been widely used in almost all oleaginous microorganisms due to their significant effectiveness. However, the signal transduction mechanisms that trigger metabolic changes in response to different stress factors are very poorly understood. ROS signaling may act as a mediator in cellular responses to extracellular environmental stresses. As a quantitative marker of cellular stress, increased ROS levels have been frequently reported in association with lipid overproduction. Here, we list the possible links among environmental stress factors, ROS signaling, and lipid metabolism, which may provide guidance for the development of stress-based strategies to enhance microbial lipid production.

The research on ROS signaling was often hindered by the lack of quantitative, dynamic, and specific techniques to monitor different activities of ROS *in vivo* (Marschall and Tudzynski, 2014). However, a variety of specific imaging tools, including small molecule fluorescent probes and genetically encoded redox probes, have been developed in recent years, and these new methods provide improved selectivity, making them able to characterize the subcellular localization and flux of ROS (Meyer and Dick, 2010; Dikalov and Harrison, 2014). Moreover, advanced proteomics techniques, such as the “redox proteome,” have paved the way for the study of cellular redox metabolism and its tight connection with biological structure and function (Go and Jones, 2013). Recent work showed importance of Ca^2+^ signal transduction in lipid accumulation in response to nitrogen starvation, and there may exist potential cross-talking between these two signaling pathways (Chen et al., 2014). Unraveling these complex signaling networks may provide guidance for the development of new stress-based strategies for enhanced microbial lipid overproduction.

## Author Contributions

KS and XJ-J wrote the manuscript. ZG, T-QS, PS, L-JR, and HH revised the manuscript.

## Conflict of Interest Statement

The authors declare that the research was conducted in the absence of any commercial or financial relationships that could be construed as a potential conflict of interest.
